# Anti-Itching and Anti-Inflammatory Effects of Kushenol F via the Inhibition of TSLP Production

**DOI:** 10.3390/ph15111347

**Published:** 2022-10-31

**Authors:** Seongyea Jo, Eun-Yeung Gong, Wonbeak Yoo, Hyunji Choi, Dana Jung, Kyung Hee Noh, Seokho Kim, Sang-Hyun Kim, Hyeong-Kyu Lee

**Affiliations:** 1 Industrial Bio-Materials Research Center, Korea Research Institute of Bioscience and Biotechnology, Daejeon 34141, Korea; 2 Laboratory of Stem Cell and Tissue Generation, Department of Biotechnology, College of Life Sciences and Biotechnology, Science Campus, Korea University, Seoul 02481, Korea; 3 Department of Medicinal Biotechnology, College of Health Sciences, Dong-A University, Busan 49315, Korea; 4 Environmental Disease Research Center, Korea Research Institute of Bioscience and Biotechnology, Daejeon 34141, Korea; 5 Korea Research Institute of Bioscience and Biotechnology, Daejeon 34141, Korea; 6 Cell & Matrix Research Institute, School of Medicine, Kyungpook National University, Daegu 41944, Korea; 7 Department of Pharmacology, School of Medicine, Kyungpook National University, Daegu 41944, Korea; 8 Natural Medicine Research Center, Korea Research Institute of Bioscience and Biotechnology, Cheongju 28116, Korea

**Keywords:** kushenol F, atopic dermatitis, thymic stromal lymphopoietin (TSLP), itching, inflammation

## Abstract

Atopic dermatitis (AD) is a chronic inflammatory skin disease that results from eczema, itching, disrupted barrier function and aberrant cutaneous immune responses. The aim of the present study was to assess the efficacy of kushenol F as an effective treatment for AD via the suppression of thymic stromal lymphopoietin (TSLP) production. The results of the present study demonstrated that the clinical symptoms of AD were less severe and there was reduced ear thickening and scratching behavior in kushenol F-treated *Dermatophagoides farinae* extract (DFE)/1-chloro-2,4-dinitrochlorobenzene (DNCB)-induced AD mice. Histopathological analysis demonstrated that kushenol F decreased the DFE/DNCB-induced infiltration of eosinophil and mast cells and TSLP protein expression levels. Furthermore, kushenol F-treated mice exhibited significantly lower concentrations of serum histamine, IgE and IgG2a compared with the DFE/DNCB-induced control mice. Kushenol F also significantly decreased phosphorylated NF-κB and IKK levels and the mRNA expression levels of IL-1β and IL-6 in cytokine combination-induced human keratinocytes. The results of the present study suggested that kushenol F may be a potential therapeutic candidate for the treatment of AD via reducing TSLP levels.

## 1. Introduction

Atopic dermatitis (AD) is a chronic inflammatory skin disease characterized by severe itching and recurrent eczema lesions [1]. AD is caused by the complex interactions of various factors, including skin barrier dysfunction, an abnormal immune response, exposure to various allergens and genetic factors [2]. The onset of AD occurs via two mechanisms: (i) Primarily, the dysfunction of the skin barrier; and (ii) an abnormal immune response [3]. Moreover, the pathogenesis of AD is caused by the interaction between various cells and molecular mediators [4,5]. Scratching due to itching aggravates skin irritation caused by secondary infections at the lesion site. Therefore, itching relief is one treatment option for AD, whereby the development of chronicity is inhibited.

Thymic stromal lymphopoietin (TSLP) is a novel IL-7-like T-helper 2 (Th2) cell-promoting cytokine, which is produced by numerous cell types, including keratinocytes [6,7]. TSLP is an important immunologic factor in AD pathogenesis via mature dendritic cells, which enhances the differentiation of naïve CD4^+^ T-cells into inflammatory Th2 cells [8]. TSLP serves a key role in the hyperactive immune response in the skin and triggers itching via directly activating sensory neurons [9,10,11,12,13]. Therefore, inhibition of TSLP production may be a potential novel therapeutic approach to treating AD via the suppression of both the hyperactive immune response and itching. Steroids and calcineurin inhibitors have widely been used in the treatment of AD; however, chronic usage excessively suppresses the immune response and causes side effects [14,15,16]. Moreover, antibodies targeting TSLP have been actively developed as therapeutics but the mass production of antibodies is expensive. Alternatively, numerous natural compounds have exhibited anti-inflammatory properties and have the potential to treat inflammatory skin diseases, especially AD [17,18,19]. These herbal extracts are not only mass-extracted, but also significantly reduce development costs. In traditional medicine, numerous compounds of *Sophora flavescens* have been clinically used in numerous countries, including China, Japan, Korea, India and certain European countries for the treatment of AD. Dried *S. flavescens* is a well-known herbal medicine and is widely used in the clinical treatment of viral hepatitis, cancer, gastrointestinal hemorrhage and skin diseases [20,21,22]. In spite of various studies regarding the biological effects of *S. flavescens* or the diverse extracts of *S. flavescens* roots, the anti-AD effect of *S. flavescens* is unclear.

Kushenol F is a flavanonol that has been isolated from *S. flavescens* roots [23]. Kushenol F has been reported to have anti-tumor, anti-inflammatory and anti-bacterial properties through suppression of MAPK-related pathways [24,25,26]. Therefore, the aim of the present study was to investigate the effects of kushenol F on a *Dermatophagoides farinae* extract (DFE)/1-chloro-2,4-dinitrobenzene (DNCB)-induced AD mouse model and cytokine combination (CC)-induced cell model. This investigation was performed to assess the potential efficiency and underlying mechanisms of kushenol F in the treatment of AD.

## 2. Results

### 2.1. Inhibitory Effect of Kushenol F on TSLP Levels in NHEKs and HSEMs

**Figure 1:**
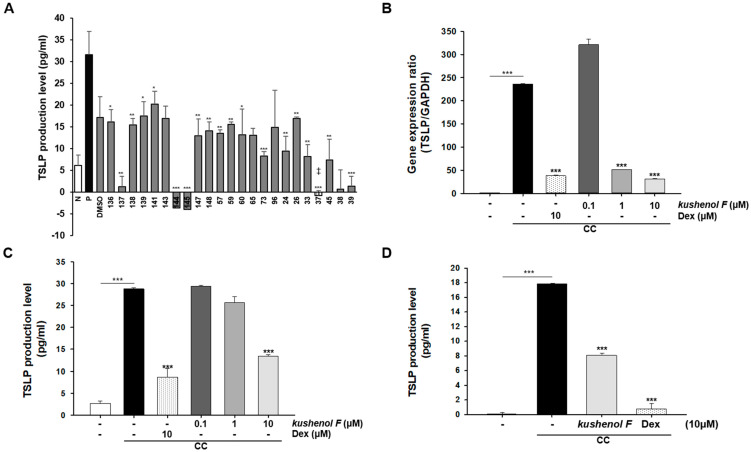
Screening for inhibitor of TSLP production and expression. (**A**) Pre-screening results of 23 natural compounds. TSLP production was determined following 24 h of treatment. NHEKs were pretreated with kushenol F for 1 h and were subsequently stimulated with CC for 4 h. (**B**) mRNA expression levels of TSLP were analyzed using reverse transcription-quantitative PCR and (**C**) TSLP concentration was quantified using ELISA. (**D**) HSEMs were pretreated with kushenol F for 1 h and then stimulated with CC for 4 h. TSLP concentration was quantified using ELISA. Data are presented as the mean ± SD of three independent experiments. ‡, kushenol F. * *p* < 0.05, ** *p* < 0.01 and *** *p* < 0.001 vs. control. TSLP, thymic stromal lymphopoietin; NHEK, normal human epithelial cells; CC, cytokine-combination; HSEM, human skin-equivalent model.

It was previously determined via a pre-screening assay, which was for the assessment of CC-induced TSLP expression in NHEKs at 10 μM concentration against 23 natural compounds from several plants, that compound 37 (kushenol F) would be selected for further study as one of the active compounds. This was isolated from the roots of *S. flavescens* (Figure 1A) [23]. First, the cell viability with kushenol F was assessed via the MTT assay. NHEKs were exposed to various concentrations of kushenol F for 24 h. Only the highest dose (50 µM) of kushenol F resulted in significant differences in cell viability compared with the control cells treated with DMSO or Dex (Figure S1). Subsequently, to examine whether kushenol F inhibited TSLP production, NHEKs were pretreated with kushenol F for 1 h prior to stimulation with CC. In the cytokine combination (CC) experiment, we added a cytokine combination for induction of TSLP overexpression and AD condition [27]. At 24 h, the mRNA expression levels and TSLP concentration were elevated in CC-treated NHEKs. However, kushenol F treatment reduced both mRNA and protein expression levels significantly (Figure 1B,C). Moreover, to verify the results in NHEKs, a well-established physiological relevant platform, HSEMs, was used. Similar to the observations in NHEKs, TSLP levels were significantly reduced in kushenol F treated HSEMs. These data indicated that kushenol F effectively inhibited CC-induced TSLP production and mRNA expression in a human keratinocyte model.

### 2.2. Anti-AD Effects of Kushenol F in the DFE/DNCB-Induced Mouse Model

**Figure 2:**
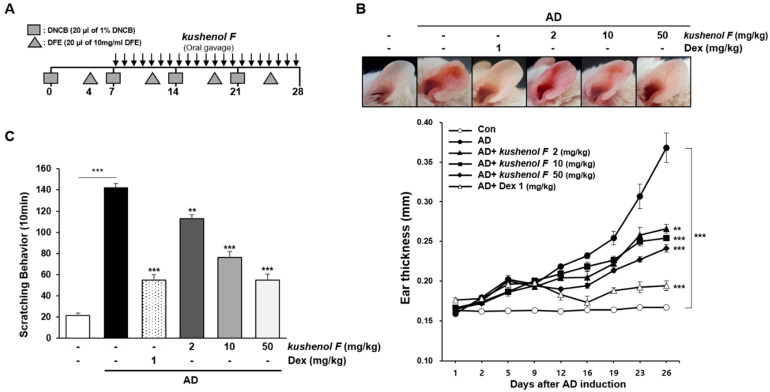
Kushenol F ameliorates clinical symptoms in AD mice. (**A**) Schematic diagram of the present study. DNCB was administered first and DFE was used 4 days later on each ear. DNCB or DFE treatment was repeated once a week rotationally for 4 weeks. After 1 week of induction, kushenol F (2, 10 or 50 mg/kg) was orally administered daily for 3 weeks. (**B**) Ear thickness was assessed 24 h following DFE or DNCB application with a dial thickness gauge. Representative photographed examples of the effects of kushenol F on DFE/DNCB-induced AD-like skin lesions in the ear. (**C**) Mouse scratching behavior of was assessed 4 h following the last sensitization for 10 min. Data are presented as the mean ± SD (*n* = 5). ** *p* < 0.01 and *** p < 0.001 vs. control. AD, atopic dermatitis; DFE, *Dermatophilosis farinae* extract; DNCB, 1-chloro-2,4-dinitrochlorobenzene.

To investigate the effect of kushenol F on AD-like skin lesions, kushenol F was orally administered to a DFE/DNCB-induced AD-like skin mouse model (AD mice). Surface of both ears was stripped with surgical tape and DNCB was painted on each ear. Subsequently, 4 days later, DFE was painted on each ear. DFE/DNCB treatment was repeated once a week rotationally for 4 weeks. After 1 week of induction, kushenol F was orally administered daily for 3 weeks. During the induction period, the ear thicknesses of mice were quantified. The ears of AD mice became red and swollen after 26 days, whereas AD mice that were treated with kushenol F exhibited symptom relief. Moreover, the kushenol F treatment group exhibited markedly reduced AD-induced ear thickness (Figure 2B). The body weight of the mice did not change among the other groups (data not shown). Furthermore, the severe behavior of scratching was quantified for 10 min following the final DFE/DNCB challenge. The results demonstrated that a suppressive effect of kushenol F on scratching behavior was observed (Figure 2C). These results indicated that kushenol F attenuated AD-like skin lesions in the DFE/DNCB-induced mouse model.

### 2.3. Anti-Inflammatory Effects of Kushenol F on AD-like Skin and Serum

**Figure 3:**
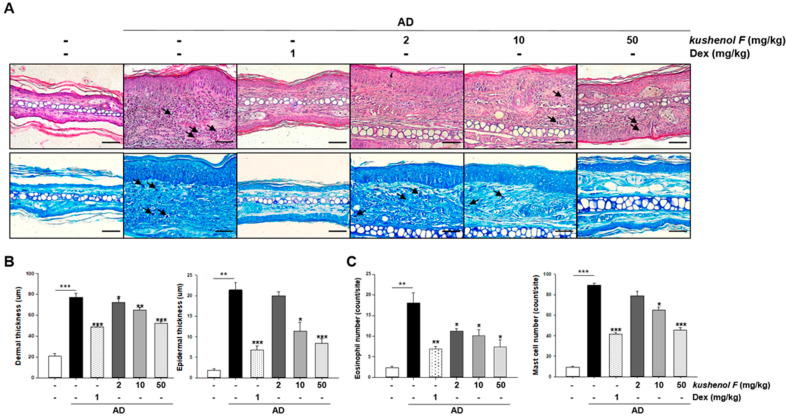
Comparison of the histopathological analysis of dorsal skin lesions in DFE/DNCB-treated atopic dermatitis mice with mice treated with kushenol F. (**A**) Representative photomicrographs of ear sections stained with H&E (upper panel arrow, eosinophil cells) or toluidine blue (lower panel arrow, mast cells). Scale bar = 10 µm. (**B**) Epidermal and dermal thickness (µm). (**C**) Number of cells is presented as the mean number of cells at five random sites for each animal. Data are presented as the mean ± SD (*n* = 5). * *p* < 0.05, ** *p* < 0.01 and *** *p* < 0.001 vs. control. DFE, *Dermatophagoides farinae* extract; DNCB, 1-chloro-2,4-dinitrochlorobenzene.

Subsequently, it was investigated whether kushenol F affected histological changes. Ear tissues were obtained from the mice and were stained with H&E or TB (Figure 3A). Histological analysis demonstrated that oral-administration of kushenol F markedly reduced dermal and epidermal thickness compared with AD-like skin lesions in ear tissues from AD mice (Figure 3B). Furthermore, the number of eosinophil and mast cells in AD-like skin lesions were significantly reduced by kushenol F treatment in a dose-dependent manner (Figure 3C).

**Figure 4:**
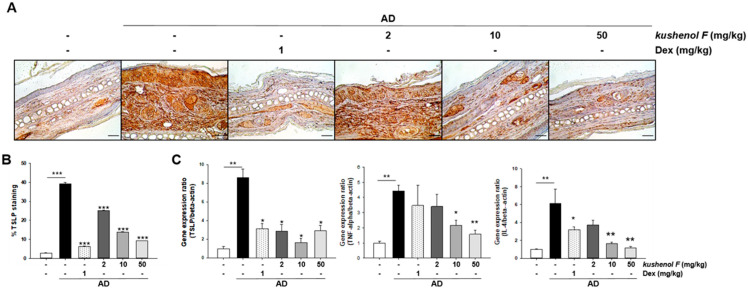
TSLP and cytokine expression analysis of dorsal skin lesions in DFE/DNCB-treated atopic dermatitis mice treated with kushenol F. (**A**) Paraffinized ear sections were examined using the TSLP antibody. Scale bar = 10 µm. (**B**) TSLP expression levels were quantified using ImageJ software. (**C**) TSLP, TNF-α and IL-4 mRNA expression levels in the ear skin of kushenol F-treated mice. Data are presented as the mean ± SD (*n* = 5). * *p* < 0.05, ** *p* < 0.01 and *** *p* < 0.001 vs. control. TSLP, thymic stromal lymphopoietin; DFE, *Dermatophagoides farinae* extract; DNCB, 1-chloro-2,4-dinitrochlorobenzene.

Epithelial cell-derived TSLP secretion promotes itch signaling [4]. Kushenol F-treated mice exhibited significantly reduced scratching behavior and mRNA expression levels and TSLP concentration in vitro. These results suggested that kushenol F treatment may be potentially important in reducing itch signaling via the production of less TSLP. Therefore, TSLP immunohistochemical staining and gene expression analysis in ear tissues from the AD mice was performed. The results demonstrated that there was a significant decrease of TSLP positive cells in kushenol F-treated mice (Figure 4A,B). Consistent with these findings, TSLP mRNA expression levels were decreased in the ear skin of kushenol F-treated mice (Figure 4C). Furthermore, the expression levels of pro-inflammatory cytokines, TNF-α and IL-4, were significantly reduced in kushenol F treated mice (Figure 4C). Together, these findings demonstrated that kushenol F reduced the recruitment of inflammatory cells and AD-induced TSLP expression levels as well as the mRNA expression levels of proinflammatory cytokines in vivo.

**Figure 5:**
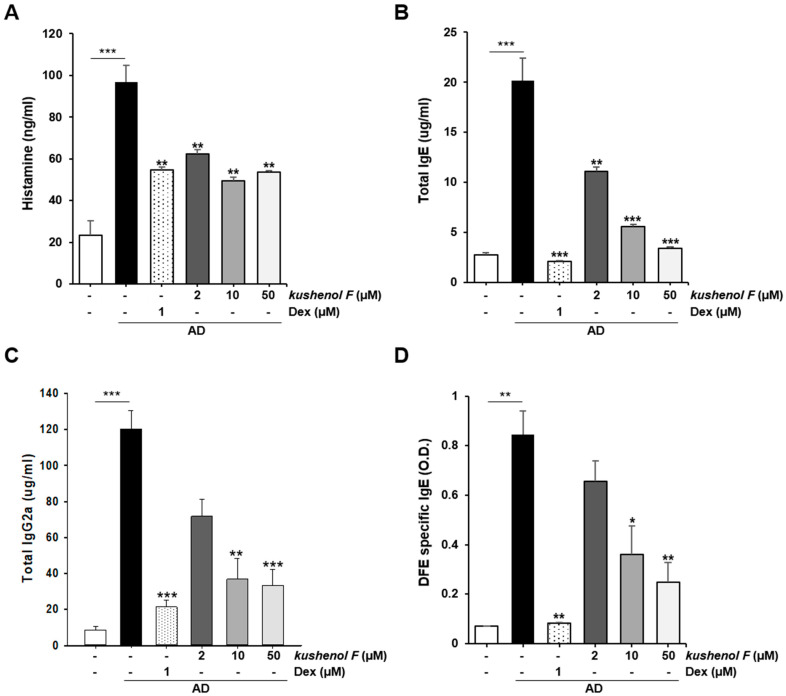
Effect of kushenol F on the serum histamine level and immunoglobulins. (**A**) Histamine levels were detected using a fluorescent plate reader. (**B**) Serum IgE, (**C**) serum IgG2a and (**D**) DFE-specific IgE concentrations were quantified using ELISA. The blood samples of the vehicle, DFE/DNCB + vehicle and DFE/DNCB + kushenol F (2, 10 or 50 mg/kg) groups were collected from the facial vein at 28 days. Data are presented as the mean ± SD (*n* = 5). * *p* < 0.05, ** *p* < 0.01 and *** *p* < 0.001 vs. control. DFE, *Dermatophagoides farinae* extract; DNCB, 1-chloro-2,4-dinitrochlorobenzene.

As histamine and IgE and IgG2a concentrations are an indicator for the development of AD in the Th1 or Th2 response these responses were also quantified in mouse serum. The results demonstrated that kushenol F-treated mice produced significantly less histamine, total IgE, DFE-specific IgE and IgG2a in a dose-dependent manner (Figure 5). These data indicated that kushenol F may potentially ameliorate AD-like skin lesions and clinical symptoms in AD mice.

### 2.4. Inhibitory Effects of Kushenol F on the Cytokine-Induced Inflammatory Response in NHEKs

**Figure 6:**
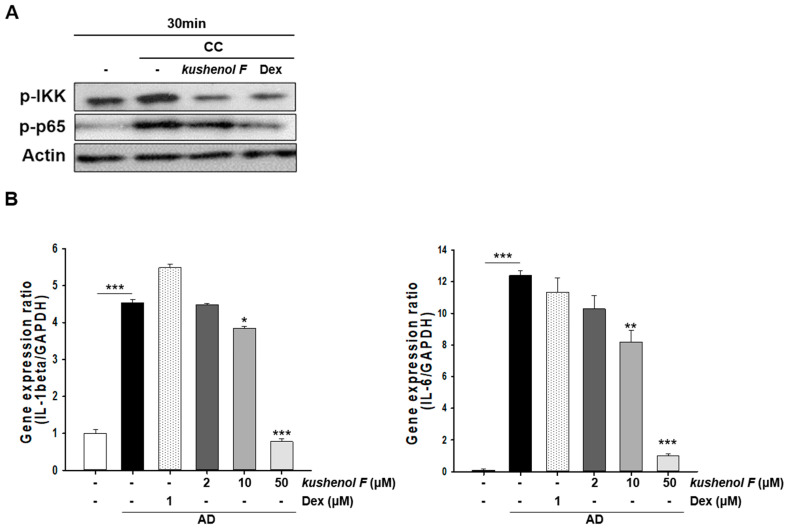
Effect of kushenol F on the inflammatory response in CC-induced NHEKs. (**A**) Effect of kushenol F on the NF-κB signaling pathways. The cells were pretreated with kushenol F (10 μM) or dexamethasone (10 μM) for 1 h and were subsequently stimulated with CC for 30 min. The cells were subjected to Western blotting. β-actin was used as a loading control. (**B**) mRNA expression levels of IL-1β and IL-6. Data are presented as the mean ± SD of three independent experiments. * *p* < 0.05, ** *p* < 0.01 and *** *p* < 0.001 vs. control. CC, cytokine combination; NHEK, normal human epithelial cell.

To assess the efficacy of kushenol F at the cellular and molecular levels, the inhibitory ability of kushenol F on inflammatory activity in CC-induced NHEKs was explored. These cells were treated with CCs to increase p-NF-kB and p-IKK. The results demonstrated that kushenol F treatment decreased p-NF-kB and p-IKK protein expression levels. These results were also similar with Dex treatment (Figure 6A). Consistent with these findings, the expression levels of IL-1β and IL-6 was significantly reduced in kushenol F-treated NHEK compared with CC-induced NHEKs in a dose-dependent manner (Figure 6B). Together, these findings demonstrated that kushenol F may reduce the inflammatory response in CC-induced NHEKs.

## 3. Discussion

For the past few decades, antihistamine agents, immunosuppressive agents or steroids have been used for the treatment of AD. However, long-term treatment causes undesirable adverse effects and drug intolerance [28,29]. Furthermore, AD is a chronic disease that occurs via a damaged skin barrier and leads to changes in local and systemic immune responses. Therefore, there have been numerous attempts to assess the therapeutic effects of the oral administration of natural substances as potential therapeutic agents [30,31]. *S. flavescens* and its metabolites have been reported to have anti-inflammatory and anti-cancer effects [32,33]. Recently, kushenol F was suggested as an immunomodulator that can improve asthma symptoms by decreasing airway-inflammation-related oxidative stress [34]. However, the effect of kushenol F, isolated from *S. flavescens,* on AD has not been investigated.

Previously, keratinocyte-derived TSLP was identified as a master regulator, which activates mast cells and eosinophils for the initiation and progression of atopic inflammation [35,36]. TSLP also serves a role in the chronicity of Th2 inflammatory responses and the stimulation of sensory neurons, which directly evokes itching behaviors, a further hallmark of atopic skin [37,38]. Furthermore, increased levels of TSLP significantly enhance the expression levels of proinflammatory cytokines, IL-6 and IL-1β, whereas the inhibition of TSLP expression reduces the infiltration of Th2 inflammatory cells, inflammatory cytokines and IgE secretion [39]. TSLP can also promote Th2 cytokine responses via its effect on mast cells, innate lymphoid cells, epithelial cells, macrophages and basophils [40,41]. Therefore, recent therapeutic studies have focused on TSLP regulation. Tezepelumab (AMG 157/MEDI9929) is a human IgG2 monoclonal antibody that acts against TSLP and prevents its interaction with the TSLP-receptor complex [42]. With TSLP inhibition, type 2 inflammation decreases, the asthma exacerbation rate is significantly reduced and clinical symptoms are improved. Therefore, inhibition of the TSLP signaling pathway has the potential to exert significant clinical effects on allergic diseases. Moreover, Venkataramani et al. [43] developed two bispecific antibodies (Zweimab and Doppelmab) that are utilized for targeting both TSLP and IL-13. These aforementioned studies are consistent with the findings of the present study, in which kushenol F decreased ear swelling, epidermal hyperplasia and the infiltration of dermal eosinophils and mast cells in ear tissues from DFE/DNCB-induced AD mice. Furthermore, the expression levels of Th2 cytokines, such as IL-4 and TNF-α, were significantly decreased in kushenol F-treated AD-lesioned ear tissue. Kushenol F also decreased the concentration and mRNA expression levels of TSLP as well as itching behavior. Collectively, these results suggested that kushenol F ameliorated the clinical symptoms of AD in DFE/DNCB-induced AD mice, which may potentially be derived from the downregulation of TSLP.

**Figure 7:**
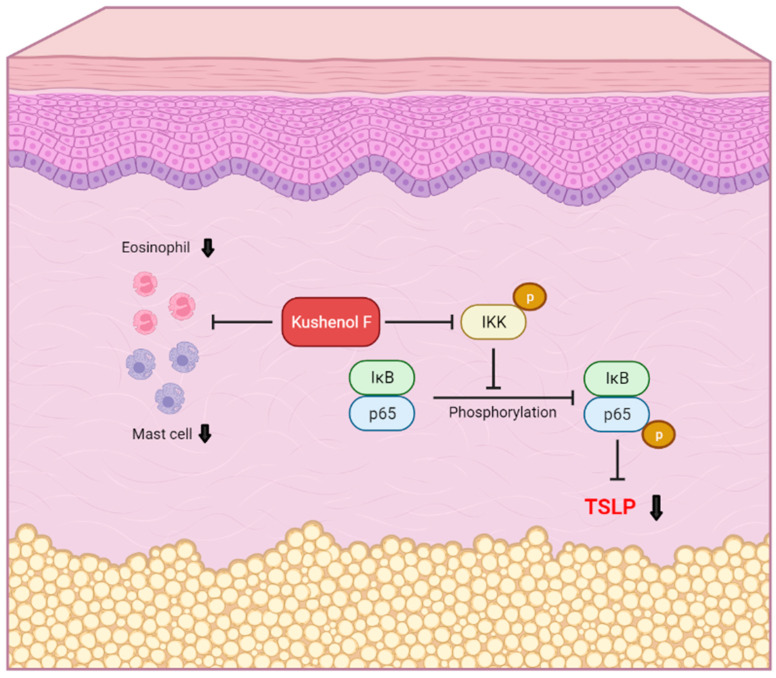
Summary diagram of kushenol F signaling pathway of this study.

NF-κB is a well-known master regulator of proinflammatory gene expression, including of IL-1β and TNF-α [44]. Previous studies have reported that the production of TSLP is dependent on the NF-kB signaling pathway [45,46,47]. Moreover, TNF-α and Th2 cytokines can promote TSLP production in ex vivo tissues and in vitro cultures of epithelial cells [48,49]. In the present study, the results demonstrated that kushenol F treatment decreased the phosphorylation of NF-κB and the mRNA expression levels of the proinflammatory cytokines, IL-1β and IL-6. The elevated expression levels of TNF-α and Th2 cytokines were decreased by kushenol F treatment, which suggested that kushenol F may help to reduce TSLP production, the infiltration of immune cells and itching behavior, which may potentially synergistically alleviate cutaneous inflammation in AD-like skin lesions (Figure 7). Further study is necessary to clarify the precise underlying mechanisms of kushenol F on the upstream signaling of TSLP.

In summary, the present study indicated that kushenol F oral treatment in DFE/DNCB-induced AD mice may markedly reduce typical symptoms. In an animal model, it was demonstrated that kushenol F treatment significantly alleviated DFE/DNCB-induced increases in skin lesion severity, ear thickness, scratching behavior and TSLP expression. Furthermore, the DFE/DNCB-induced infiltration of eosinophils and mast cells was decreased following kushenol F treatment. The present study also determined that kushenol F treatment lowered levels of serum histamine, IgE and IgG2a in DFE/DNCB-treated AD mice. Therefore, the present study demonstrated that kushenol F oral treatment may potentially be an important therapeutic for the treatment of AD.

## 4. Materials and Methods

### 4.1. Preparation of Plant Extracts

Kushenol F (C_25_H_28_O_6_; molecular weight, 424.5) was purchased from the Korea Plant Extract Bank [50] and identified via instrumental analysis methods, including 1D- and 2D-nuclear magnetic resonance spectroscopy (NMR) and liquid chromatography/mass spectrometry. Its purity was >98% as determined via quantitative NMR analysis. For in vitro and in vivo experiments, kushenol F was dissolved in DMSO (Sigma-Aldrich; Merck KGaA, St. Louis, MO, USA) and diluted with assay media or PBS.

### 4.2. Animals

Female BALB/c mice (age, 6 weeks) were purchased from Japan (SLC, Inc., Hamamatsu, Japan). The mice were maintained in a laminar air flow room at a temperature of 22 ± 2 °C, a relative humidity of 55 ± 5% and in 12 h light/dark cycles throughout the experiment. For anesthesia 3% ether was used and for euthanasia CO_2_ was used at ~70% vol/min according to the weight of mouse. All animal housing complied with and all experiments were performed in accordance with the Institutional Animal Care and Use Committee of Kyungpook National University (# KNU 2017-0007) Guidelines.

### 4.3. Induction of AD-like Lesions in the Ears of Mice

AD-like lesions were induced using DFE (Greer Laboratories, Inc., Lenoir, NC) and DNCB according to previous studies [51]. DFE was dissolved in PBS containing 0.5% Tween-20 and 1% DNCB, which was then mixed with acetone/olive oil (1:3) solution. The schematic for this experimental procedure is presented in Figure 2A. A total of 30 female BALB/c mice were divided into the following six groups (*n* = 5/group): (i) Vehicle; (ii) DFE/DNCB + vehicle; (iii) DFE/DNCB + kushenol F (2 mg/kg); (iv) DFE/DNCB + kushenol F (10 mg/kg); v) DFE/DNCB + kushenol F (50 mg/kg); and (vi) dexamethasone (Dex; 1 mg/kg). Mice were anesthetized using ether and the surface of both ears was stripped using surgical tape four times (Nichiban Co., Tokyo, Japan). Subsequently, 20 μL 1% DNCB was painted on each ear and 4 days later, 20 μL DFE (10 mg/mL) was painted on each ear. DFE and DNCB treatment was repeated once a week rotationally for 4 weeks. Kushenol F was orally administered during the induction period for 3 weeks. Ear thickness was analyzed the following day at the same time following DFE or DNCB application using a dial thickness gauge (Mitutoyo, Tokyo, Japan). The health and behavior of the mice were monitored daily and no animals died during the experiments. The subsequent humane endpoints were used: (i) Walking uncomfortably and consuming food and water; (ii) Trouble maintaining a normal posture due to weakness; (iii) Decrease in weight of >20% compared with the control group; (iv) Coarse breathing, cyanosis, chronic discomfort or constipation; (v) Hematological or blood biochemistry parameters indicating organ function decline that compromises survival ability; (vi) falling into an unconscious state and not responding to external stimuli. On the last day of the 4 weeks experiment, the mice were deeply anesthetized with CO_2_ at ~70% vol/min according to the weight of mouse. Absence of a corneal reflex, failure to detect respiration, and absence of a heart beat for a period of more 5 min used to confirm death. Then, blood samples were collected via orbital puncture. After the blood had clotted at room temperature it was centrifuged at 400× *g* for 15 min at 4 °C and the serum was isolated. The serum was stored at −80 °C for additional analysis. The ear of each mouse was removed for histopathological analysis.

### 4.4. Scratching Behavior

The animals were placed into an observation cage for 10 min for acclimatization prior to the assessment of scratching behavior. The quantity of scratching behaviors was counted for 10 min each time.

### 4.5. Histological Examination

The ears were fixed using 10% formaldehyde and embedded in paraffin. Sections (9 μm) were stained using H&E and toluidine blue (TB). For immunohistochemistry, the TSLP antibody (cat. no. ab188766; Abcam, Cambridge, UK) was used. The epidermal and dermal thicknesses in five randomly selected fields from each sample (magnification, 400×) were assessed using an optical microscope (CX21; Olympus Corporation, Tokyo, Japan) and images were analyzed using ImageJ (version 1.8.0_112; National Institutes of Health, Bethesda, MA, USA). The number of eosinophils and mast cells at five random sites were quantified (magnification, 400×). Eosinophils and mast cells were also quantified using ImageJ.

### 4.6. Cell Culture and Stimulation of Keratinocytes and the 3D Skin Model

Normal human epidermal keratinocytes (NHEKs) were purchased from MilliporeSigma and cultured for 3–4 passages using the EpiGRO™ Human Epidermal Keratinocyte Complete Media Kit (MilliporeSigma, Burlington, MA, USA). Human skin-equivalent models (HSEMs) were purchased from MetTek. HSEMs were cultivated according to the manufacturer’s instructions. NHEKs were grown in a humidified atmosphere containing 5% CO_2_ and 95% air atmosphere. Cells were seeded at a density of 2 × 10^4^ cells/well in a flat-bottomed 96-well microculture plate and were cultured until they reached 90% confluence, then the media was changed to fresh medium without hydrocortisone. After further cultivation for 24 h, keratinocytes were stimulated using previously described methods with CCs [52,53] that included: TNF-α (20 ng/mL, R&D system), IL-4 (100 ng/mL, R&D system), IL-13 (100 ng/mL, R&D system) and flagellin (100 ng/mL; Peprotech, Inc., Seoul, Korea). Kushenol F pretreatment was performed for 1 h before keratinocyte activation.

### 4.7. Cell Viability

To assess the effect of kushenol F on cell viability, the cells were seeded at 2 × 10^4^/well in a flat-bottomed 96-well microculture plate. Cells were cultivated for 24 h following treatment with kushenol F. MTT solution (5 mg/mL) was added and the cells were incubated at 37 °C for an additional 4 h. After washing out the supernatant, the insoluble formazan product was dissolved using DMSO. Then, optical density was assessed using an ELISA reader at 570 nm.

### 4.8. Reverse Transcription (RT)-Quantitative (q)PCR

Total cellular RNA was isolated from cells using TRIzol^®®^ reagent (Invitrogen; Thermo Fisher Scientific, Inc., Waltham, CA, USA) according to the manufacturer’s protocol. Subsequently, 1 μg total RNA was used to form complementary DNA using an RT kit (BioFact Co., Ltd., Daejeon, Korea) using reverse transcriptase. qPCR was performed using Applied Biosystems (Thermo Fisher Scientific, Inc., Waltham, MA, USA) according to the manufacturer’s protocol. The qPCR thermocycling conditions were as follows: 30–35 cycles of 30 s at 95 °C, 30 s at 50–60 °C and 30 s at 72 °C. The relative mRNA expression levels were normalized using GAPDH as an internal control.

### 4.9. ELISA

TSLP concentration was quantified using an ELISA kit (R&D Systems, Inc., Minneapolis, MN, USA) according to the manufacturer’s protocol.

### 4.10. Biochemical Analysis

Serum histamine concentrations were assessed using the o-phthaldialdehyde spectrofluorometric procedure from a previous study [54]. Blood from mice was centrifuged at 400× *g* for 15 min and the serum was diluted using PBS. Fluorescence intensity was assessed at an excitation wavelength of 355 nm using 450 nm filters. A fluorescence spectrometer was used (LS-50B; PerkinElmer, Inc., Norwalk, CT, USA). Serum IgE and IgG2a concentrations were quantified using ELISA kits (BD Biosciences, Oxford, UK) according to the manufacturer’s protocol. For the detection of DFE-specific IgE, 96-well plates (Nunc; Thermo Fisher Scientific, Inc., Waltham, MA, USA) were coated with 10 mg DFE in PBS. The DFE specific IgE level was indicated by the optical density value.

### 4.11. Western Blotting

Total protein was extracted using RIPA buffer (Pierce; Thermo Fisher Scientific, Inc., Waltham, MA, USA) and protein concentration was determined using a Bradford Protein Assay kit (Bio-Rad Laboratories, Inc., Hercules, CA, USA). Total protein (30 μg/lane) was separated using SDS-PAGE and separated proteins were transferred onto PVDF membranes (MilliporeSigma, MA, USA). Membranes were blocked using 5% blocking buffer [5% *w*/*v* skimmed milk, 0.1% Tween-20 with TBS (pH 7.4)] at room temperature for 1 h. Subsequently, membranes were incubated with the following primary antibodies (1:1000) against phosphorylated (p)-NF-κB (cat. no. 3033; Cell Signaling Technology, Inc., Beverly, MA, USA) and p-IKKα/β (cat. no. 2697; Cell Signaling Technology, Inc.) at 4 °C overnight. Following primary incubation membranes were incubated with the following secondary antibodies (1:3000 dilution) against HRP-conjugated anti-rabbit IgG (cat. no. sc-2004; Santa Cruz Biotechnology, Inc., Santa Cruz, CA, USA) and HRP-conjugated anti-mouse IgG (cat. no. sc-2005; Santa Cruz Biotechnology, Inc., CA, USA) at room temperature for 1 h. Detection was performed using an ECL detection kit (Thermo Fisher Scientific, Inc., Waltham, MA, USA). β-actin (cat. no. sc-47778; Santa Cruz Biotechnology, Inc., CA, USA) was used as the loading control.

### 4.12. Statistical Analysis

GraphPad Prism 5 (GraphPad Software, Inc., San Diego, CA, USA) was used for statistical analysis. Drug treatment effects were analyzed using a one-way ANOVA followed by Dunnett’s test. A *p* *<* 0.05 was considered to indicate a statistically significant difference.

## Data Availability

Data is contained within the article and supplementary material.

## Supplementary Materials

The following supporting information can be downloaded at: https://www.mdpi.com/article/10.3390/ph15111347/s1, Figure S1: NHEKs were treated with various concentrations of kushenol F (0.1–50 μM). Cell viability was analyzed using the MTT assay. Data are presented as the mean ± SD of three independent ex-periments. * *p* < 0.05 and *** *p* < 0.001 vs. control. NHEK, normal human epithelial cell.
