# Supplementary material for: Anti-Itching and Anti-Inflammatory Effects of Kushenol F via the Inhibition of TSLP Production
Source: Pharmaceuticals (Basel). 2022 Oct 31;15(11):1347. doi: 10.3390/ph15111347 (PMC9694767; doi:10.3390/ph15111347)
Supplement: Supplementary file 1 [file pharmaceuticals-15-01347-s001.zip › pharmaceuticals-1940325-supplementary.pdf]

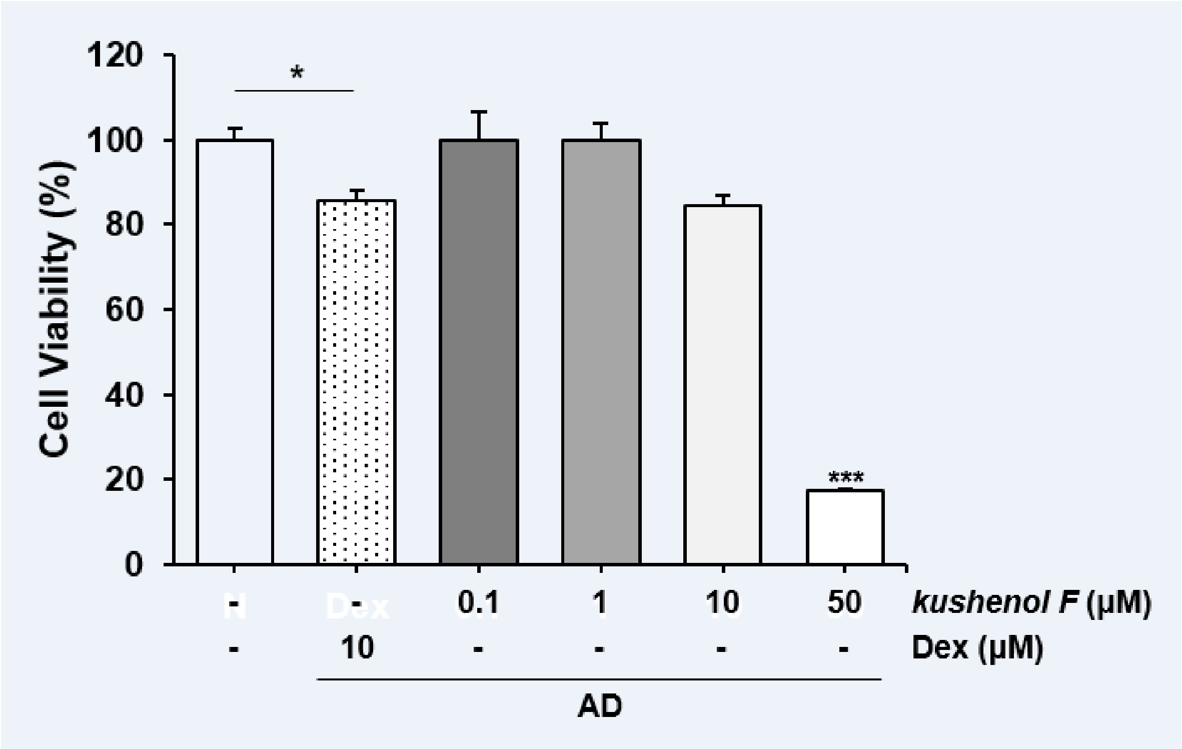

Figure S1. NHEKs were treated with various concentrations of kushenol F (0.1–50 μM). Cell viability was analyzed using the MTT assay. Data are presented as the mean ± SD of three independent ex-periments. * $p < 0.05$ and *** $p < 0.001$ vs. control. NHEK, normal human epithelial cell.
